# Antibacterial and Antivirulence Activity of Glucocorticoid PYED-1 against *Stenotrophomonas maltophilia*

**DOI:** 10.3390/antibiotics9030105

**Published:** 2020-03-02

**Authors:** Anna Esposito, Adriana Vollaro, Eliana Pia Esposito, Daniele D’Alonzo, Annalisa Guaragna, Raffaele Zarrilli, Eliana De Gregorio

**Affiliations:** 1 Department of Chemical Sciences, University of Naples Federico II, Via Cintia, 80126 Naples, Italy; anna.esposito5@unina.it (A.E.); annalisa.guaragna@unina.it (A.G.); 2 Department of Molecular Medicine and Medical Biotechnology, University of Naples Federico II, Via S. Pansini 5, 80131 Naples, Italy; vollaroadriana@libero.it; 3 Department of Public Health, University of Naples “Federico II”, Via S. Pansini 5, 80131 Naples, Italy; elianapia.esposito@unina.it (E.P.E.); rafzarri@unina.it (R.Z.)

**Keywords:** antimicrobial activity, deflazacort (DFZ), *S. maltophilia*, quantitative real-time PCR, anti-virulence agent, checkerboard assay

## Abstract

*Stenotrophomonas maltophilia*, an environmental Gram-negative bacterium, is an emerging nosocomial opportunistic pathogen that causes life-threatening infections in immunocompromised patients and chronic pulmonary infections in cystic fibrosis patients. Due to increasing resistance to multiple classes of antibiotics, *S. maltophilia* infections are difficult to treat successfully. This makes the search for new antimicrobial strategies mandatory. In this study, the antibacterial activity of the heterocyclic corticosteroid deflazacort and several of its synthetic precursors was tested against *S. maltophilia*. All compounds were not active against standard strain *S. maltophilia* K279a. The compound PYED-1 (pregnadiene-11-hydroxy-16α,17α-epoxy-3,20-dione-1) showed a weak effect against some *S. maltophilia* clinical isolates, but exhibited a synergistic effect with aminoglycosides. PYED-1 at sub-inhibitory concentrations decreased *S. maltophilia* biofilm formation. Quantitative real-time polymerase chain reaction (RT-qPCR) analysis demonstrated that the expression of biofilm- and virulence- associated genes (*StmPr1*, *StmPr3*, *sphB*, *smeZ*, *bfmA*, *fsnR*) was significantly suppressed after PYED-1 treatment. Interestingly, PYED-1 also repressed the expression of the genes *aph* (3′)-IIc, *aac* (6′)-Iz, and *smeZ*, involved in the resistance to aminoglycosides.

## 1. Introduction

*Stenotrophomonas maltophilia* is an emerging opportunistic bacterium, which represents the third Gram-negative responsible for nosocomial infections [1,2,3]. *S. maltophilia* generally causes bacteremia and pneumonia, and infections are frequently associated with complications and death in immunosuppressed or immunocompromised patients [4,5]. *S. maltophilia* is frequently found in polymicrobial infections from the respiratory tract of cystic fibrosis (CF) patients [6]. In CF patients, *S. maltophilia* infections are associated with a severe lung disease and increased risk of the need for transplantation, or death [7,8]. Many virulence factors that may contribute to the pathogenicity of *S. maltophilia* have been reported [9,10,11,12,13,14,15]. The therapeutic treatment is hampered by intrinsic or acquired resistance to multiple antimicrobial agents [16,17]. *S. maltophilia* is intrinsically resistant to carbapenems, aminoglycosides, and tetracyclines [3,16] owing to its multidrug efflux pumps and overexpression of resistant determinants, such as carbapenemases and aminoglycoside-modifying enzymes [4,16,17]. Trimethoprim–sulfamethoxazole (STX) is the first-line antimicrobial combination for the treatment of *S. maltophilia* infections. However, the allergenic reaction, the intolerance, and the increasing rates of resistance limit the use of Trimethoprim–sulfamethoxazole [18,19]. *S. maltophilia* infections are also difficult to treat due to the ability of this bacterium to form highly structured and multilayered biofilms [20,21,22]. Because of the potential for resistance development, there is need of novel antimicrobials for *S. maltophilia* infection treatment. 

Deflazacort (DFZ) is a heterocyclic corticosteroid derivative of prednisolone, used as an anti-inflammatory and immunosuppressant, and characterized by high efficacy and good tolerability [23,24]. Recently, the DFZ synthetic precursor PYED-1 (pregnadiene-11-hydroxy-16α,17α-epoxy-3,20-dione-1) showed a good antibacterial activity against *Staphylococcus aureus* ATCC 29213 and *Acinetobacter baumannii* ATCC 17978 without showing cytotoxicity [25].

The aim of this study was to evaluate the antimicrobial, antibiofilm, and anti-virulence activities of PYED-1 against *S. maltophilia*.

## 2. Results and Discussion

### 2.1. Antimicrobial Activity of a Panel of Steroid Derivatives

**Figure 1:**
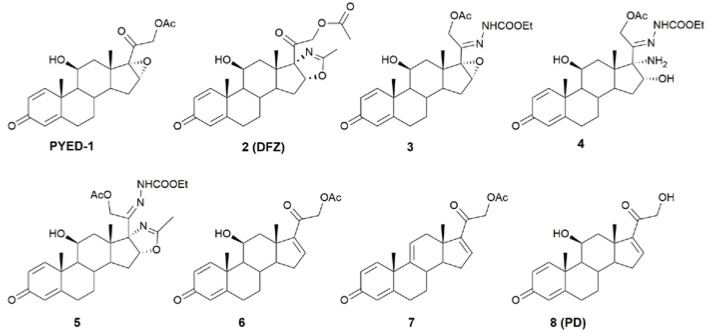
Deflazacort (DFZ), prednisolone (PD), and DFZ synthetic precursors used in this study.

The antimicrobial activity of glucocorticoid DFZ and its synthetic precursors (Figure 1) against *S. maltophilia* K279a was assessed by broth microdilution assay (Table 1).

All compounds were inactive against *S. maltophilia* K279a cells (Table 1). Instead, *S. maltophilia* K279a was susceptible to STX standard compound (Table 1). PYED-1, the compound that proved to be the most effective growth inhibitor against *S. aureus* ATCC 29213 and *A. baumannii* ATCC 17978 [25], showed MIC value at 256 μg/mL against *S. maltophilia* K279a, while MBC value at twofold upper MIC value (512 μg/mL). To examine whether the inhibition of bacterial growth could be related to the dimethyl sulfoxide (DMSO) used to dissolve the compounds tested, the growth of *S. maltophilia* K279a was measured in the presence of increasing concentrations of DMSO (ranging from 0.1% to 1%). Results indicated that there was no difference in *S. maltophilia* K279a growth in the presence of any of the DMSO concentrations used (data not shown). The antimicrobial activity of PYED-1 was also tested on a panel composed by eleven clinical isolates of *S. maltophilia* (Table 2). STX standard compound retained its activity against all *S. maltophilia* clinical isolates (data not shown).

The MIC values ranging from 64 μg/mL to 512 μg/mL. MBC values were always at least twofold higher than MIC values (see Table 2). The killing quotient (KQ), which corresponds to the MBC/MIC ratio, indicated that the compound has bactericidal (≤4) or bacteriostatic (>4) activity [26]. As shown in Table 2, KQ was less than or equal to 4 for all strains, indicating that PYED-1 exhibited bactericidal activity. 

**Figure 2:**
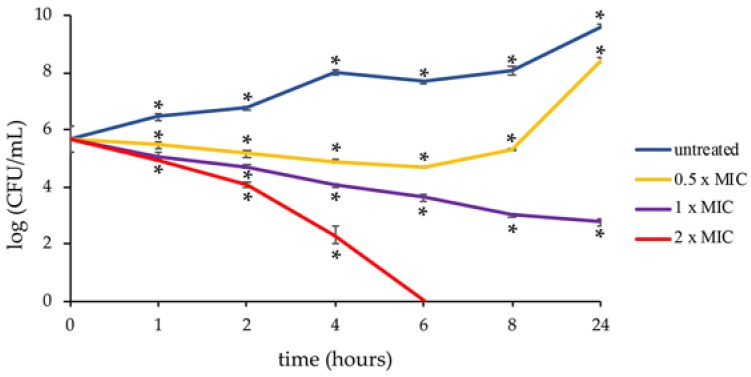
Killing kinetics for *S. maltophilia* K279a following treatment with the PYED-1. Growth kinetics were monitored following exposure to PYED-1 at 0.5 × MIC (128 μg/mL), 1 × MIC (256 μg/mL), and 2 × MIC (512 μg/mL). Values are presented as mean ± SD. *P < 0.05.

The time–kill assay showed that PYED-1 exhibited a significant bactericidal activity (Figure 2). 

At 0 h treatment, the colony forming unit (CFU) was approximately 6 × 10^5^ /mL. After 2 h of incubation with 128 μg/mL, 256 μg/mL and 512 μg/mL, the bacterial load was reduced approximately five, ten, and fifty times, respectively. Following 4 h exposure with the indicated MIC, CFU declined to 8 × 10^4^, 1 × 10^4^, and 2 × 10^2^, respectively. No viable *S. maltophilia* cells were recovered after six hours’ exposure at 2 × MIC. However, after 24 h the cell growth recovery was observed at 128 μg/mL. By definition, a compound that kills ≥3 log_10_ bacteria following 24 h incubation is considered bactericidal [27]. In this study, the PYED-1 MIC reduced the number of CFUs by 3.2 log_10_ following 24 h incubation, indicating that PYED-1 was bactericidal at this concentration. 

**Figure 3:**
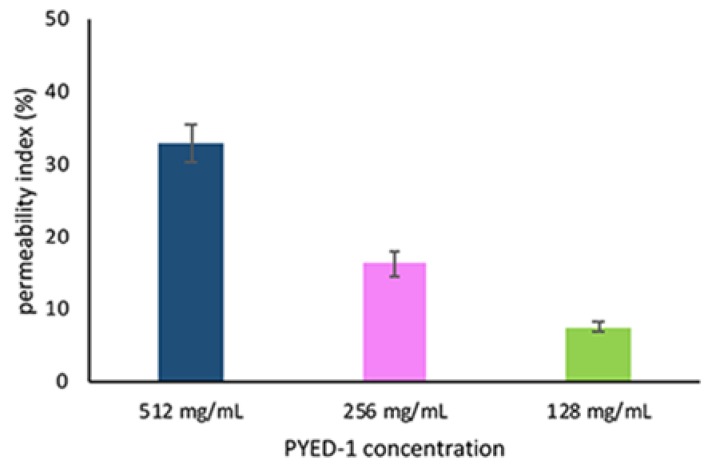
Effect of PYED-1 on *S. maltophilia* K279a membrane permeability. *S. maltophilia* K279a cells were treated with 2 × MIC (512 μg/mL), 1 × MIC (256 μg/mL) and 1/2 × MIC (128 μg/mL), and PI uptake was measured after 4 h of treatment. The error bars represent standard error of the mean from three independent experiments.

The bacterial uptake of the membrane impermeant dye propidium iodide (PI) was measured to assess the membrane permeability of *S. maltophilia* K279a following treatment with PYED-1. The increase of PI fluorescence indicated the loss of bacterial membrane integrity. As shown in Figure 3, PYED-1 increased PI uptake into *S. maltophilia* in a dose-dependent manner. PI-fluorescence intensity of *S. maltophilia* K279a cells increased by 33%, 16% and 7.5% after 4 h incubation with PYED at 2 × MIC, 1 × MIC, and 1/2 × MIC, respectively. Based on the above finding, we hypothesize that the permeabilization of the bacterial membrane might contribute to the bactericidal activity of PYED-1. Further experiments are necessary to validate this hypothesis. 

### 2.2. Checkerboard Assay

*S. maltophilia* is intrinsically resistant to aminoglycosides, a class of conventional antibiotics mostly used in the treatment of respiratory and urinary tract infections, blood, bone, and soft tissue infections caused both by Gram-negative and Gram-positive bacteria [16]. To find out whether PYED-1 combined with aminoglycosides may potentiate the antibacterial activity of these antibiotics against *S. maltophilia*, the activity of PYED-1 in combination with gentamycin and amikacin was determined using broth microdilution checkerboard assay against *S. maltophilia* K279a. The fractional inhibitory concentration (FIC) index for these combinations is reported in Table 3. 

In combination with PYED-1, MIC was remarkably reduced by eightfold (from 16 to 2 μg/mL) and fourfold (from 16 to 4 μg/mL) for gentamicin and amikacin, respectively. These results demonstrated synergistic effect with aminoglycosides. The PYED-1 concentration of 64 μg/mL (1/4 MIC) was able to give the most synergistic effect with both aminoglycosides. The synergistic effect of PYED-1 and gentamicin and amikacin is an important finding because it allows the extension of the antimicrobial strategies against multidrug-resistant *S. maltophilia* [3,4,16,17].

### 2.3. Effects of PYED-1 on the Formation of S. maltophilia Biofilm

**Figure 4:**
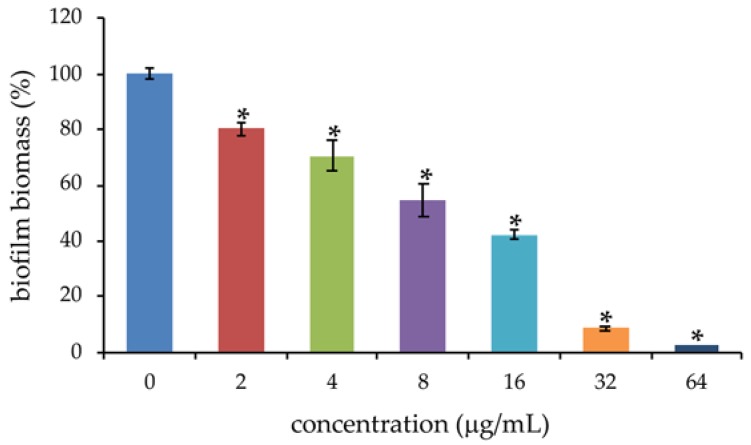
*S. maltophilia* K279a biofilm formation following treatment with the PYED-1. Cells were incubated for 24 h in the presence of sub-inhibitory concentrations of PYED-1. Biofilms were quantified after crystal-violet staining. Values are presented as mean ± SD. * P < 0.05.

Most *S. maltophilia* strains form biofilms on several biotic and abiotic surfaces, and this greatly contributes to the pathogenicity of these bacteria [20,21,22]. Thus, the antibiofilm properties of PYED-1 against *S. maltophilia* K279a were investigated. We measured the biofilm biomass of *S. maltophilia* K279a cells treated with increasing concentrations of PYED-1 in static condition at 37 °C using abiotic crystal violet staining. Sub-inhibitory concentrations of PYED-1 were able to reduce biofilm formation of *S. maltophilia* K279a compared to the untreated control (Figure 4). 

A reduction of 97%, 90%, and 57% was found at concentrations 64 μg/mL, 32 μg/mL, and 16 μg/mL, corresponding to 1/4 × MIC, 1/8 × MIC and 1/16 × MIC, respectively. To determine whether the inhibitory effect on biofilm formation was related to growth inhibition, planktonic growth was measured in same conditions used in biofilm assay. At the concentrations tested in biofilm assay, PYED-1 did not affect planktonic growth (data not shown). Several studies demonstrated that novel classes of antimicrobial compounds inhibit *S. maltophilia* biofilm formation [28,29,30,31,32,33]. In further support of this, our results showed that PYED-1 at 64 μg/mL significantly inhibited *S. maltophilia* biofilm formation on a polystyrene abiotic surface. Interestingly, the same concentration of PYED-1 has a synergistic effect with gentamicin or amikacin on bacterial growth inhibition. This is in agreement with other studies showing that *S. maltophilia* biofilm formation plays a notable role in the development of antibiotic resistance [20,21,22].

### 2.4. Transcriptional Changes Induced by PYED-1 in S. maltophilia K279a

A promising alternative strategy to treat infections caused by multidrug bacteria is anti-virulence therapy, based on the development of drugs able to specifically inhibit virulence factors [34,35]. To investigate the anti-virulence activity of PYED-1 the expression levels of a dozen known *S. maltophilia* virulence genes [9,10,11,12,13,14,15] were investigated by qRT-PCR. RNA was extracted from exponential *S. maltophilia* cells (3 × 10^8^ CFU/mL) untreated and treated at sub-MIC concentration (128 μg/mL) of PYED-1 for 3 h. No growth differences between treated and untreated cells were observed. Also, 3 h’ PYED-1 treatment slightly increased the membrane permeability of *S. maltophilia* K279a (data not shown). In this type of experiments, genes with at least a twofold difference in relative transcript levels (with a P value of <0.05) are considered significant. As shown in Table 4, the expression of most of the tested genes was affected by PYED-1 treatment. 

PYED-1 significantly decreased the gene expression of *fsnR*, encoding the orphan response regulator FsnR, involved in motility and biofilm formation. *S. maltophilia* cell motility and biofilm formation can be inhibited by a reduction of *fsnR* gene expression [36]. Furthermore, the expression of *bfmA* gene, a transcription factor that stimulates the transcription of *bfm*AK operon involved in biofilm formation [37], was significantly downregulated by PYED-1. Based on our results, we postulate that inhibition of *bfm*A and *fsnR* gene expression by PYED-1 may contribute to the reduction of biofilm formation in *S. maltophilia*. Data are in agreement with a previous study showing that *bfm*A and *fsnR* gene expression is modulated by treatment of *S. maltophilia* K279a with celastrol [30]. 

In addition, PYED-1 significantly decreased the expression of *sphB*, *StmPr1*, and *StmPr3* genes encoding for serine proteases that contribute to degradation of extracellular matrix proteins [38]. The ability of *S. maltophilia* to produce extracellular protease may contribute to *S. maltophilia* pathogenesis in the lungs of CF patients [39,40]. The major protease StmPr1 induces the death of A549 fibroblasts and IL-8 secretion by A549 cells [40]. PYED-1 treatment could reduce the release of extracellular proteases, and consequently tissue damage and inflammation in the host. 

PYED-1 significantly reduced also the gene expression of *sme*Z, *aph3′-IIc*, and *aac6′-Iz*, three genes involved in the resistance to aminoglycosides. Interestingly, the RND-type efflux encoded by *smeZ* has been demonstrated in *S. maltophilia* to contribute to aminoglycosides resistance [41], and other virulence-related functions, such as swimming, protease secretion, and biofilm formation [42]. In further support of this, we hypothesize that PYED-1 decreases antibiotic resistance in *S. maltophilia* by inhibiting the biofilm formation and the expression of multidrug efflux pumps. Future experiments will be necessary to validate the hypothesis. 

Overall, our data demonstrate that PYED-1 in combination with gentamicin or amikacin aminoglycosides shows antimicrobial activity against *S. maltophilia*. Also, PYED-1 acts as an antibiofilm drug and inhibits the expression of important biofilm and virulence genes in *S. maltophilia*, attenuating the virulence of this drug-resistant pathogen. These results make PYED-1 a promising candidate for clinical use against *S. maltophilia* infectious diseases. Although the pharmacokinetics of corticosteroids are well known [43], no information about the pharmacokinetics of the PYED-1 is available yet. Future studies will be necessary to establish the pharmacokinetics of PYED-1 and its derivatives and their potential clinical use.

## 3. Materials and Methods 

### 3.1. Chemicals and Reagents

All chemicals and solvents were purchased with the highest degree of purity (Sigma-Aldrich, Alfa Aesar, VWR) and used without further purification. The reactions were monitored by TLC (precoated silica gel plate F254, Merck) and the products were detected by exposure to ultraviolet radiation, iodine vapor, and chromic mixture. The purity of the compounds was determined by CHNS analysis and was ≥ 95% in all cases. NMR spectra were recorded on NMR spectrometers operating at 400 MHz (Bruker DRX, Bruker AVANCE) using CDCl_3_ solutions. Coupling constant values (J) were reported in Hz. Chemical synthesis and structural characterization of compounds was realized as previously reported [25].

### 3.2. Antimicrobial Activity

Strains evaluated in this study included the *S. maltophilia* K279a reference strains and eleven *S. maltophilia* clinical isolates belonging to a bacterial collection previously established. Epidemiological features of strains were in accordance to previous publications [44,45]. No ethical approval was required for the study because there was no access to patients’ data. All strains were grown on blood agar plates (TSA). Minimum inhibitory concentration (MIC) values of steroidal compounds against planktonic bacteria were examined by a broth microdilution method previously described [46]. Briefly, stock solutions of all compounds at the concentration of 50 mg/mL were made by dissolving them in DMSO. Bacterial cell suspensions were prepared at 0.5 McFarland standard using a BD PhoenixSpec™ nephelometer and were subsequently diluted in cation-adjusted Mueller–Hinton broth (CA-MHB) to approximately 5 × 10^6^ CFU/mL. One hundred microliter of bacteria (5 × 10^5^ CFU) were then added to the microtiter plates containing 100 μL of serial dilutions of steroidal compounds. Only CA-MHB was added in negative control wells. Wells with no compounds were used on each plate as positive growth control. Plates were incubated at 37 °C for 18 h under shaking (300 rpm). The optical density at 595 nm was measured by using a microplate reader (Bio-Rad Laboratories S.r.l.). The effect of different concentrations of DMSO (ranging from 0.1% to 1%) on bacteria growth kinetics was separately tested. To calculate the minimum bactericidal concentration (MBC), bacterial suspensions from MIC assay microtiter wells were diluted in PBS and spot-plated on TSA plates, and the colonies were counted after incubation at 37 °C for 18 h. The MBC was determined as the lowest concentration of substance, which produced ≥99.9% killing (≥3 log_10_) after 24 h of incubation as compared to the colony count of the starting inoculum. All tests were performed in triplicate and repeated three times. 

### 3.3. Time Killing Assay

The killing kinetics of PYED-1 at 0.5 ×, 1 × and 2 × MIC were determined against *S. maltophilia* K279a. Approximately 6 × 10^5^ CFU/mL of *S. maltophilia* K279a strain was used to inoculate 3 mL of CA-MHB containing different concentrations of PYED-1 and incubated at 37 °C under shaking (300 rpm). A tube without PYED-1 was a growth control. Viable bacterial counts were performed after 0, 1, 2, 4, 6, 8, and 24 h incubation by plating serial tenfold dilutions of broth cultures onto TSA plates, and incubating at 37 °C for 24 h. All experiments were repeated three times.

### 3.4. Propidium Iodide Uptake Assay 

Cell permeability was assessed as reported earlier [47]. Briefly, *S. maltophilia* cells were grown in CA-MHB up to the mid logarithmic phase, adjusted to 1 × 10^6^ CFU/mL and incubated at 37 °C with PYED-1 (512 μg/mL, 256 μg/mL, and 128 μg/mL) for 4 h. After PYED-1 treatment, the cells were washed in PBS buffer and incubated with PI (10 μM) at 37 °C for 20 min in the dark. Bacterial cells permeabilized with 0.5% Triton X100 were used as a positive control. Untreated bacterial cells were used as a negative control. The PI fluorescence was measured at excitation and emission of 485 nm and 590 nm respectively, using the plate reader Synergy HT spectrofluorimeter (Biotek). Fluorescence was normalized by subtracting fluorescence of the untreated cells from that of the treated. Permeability index was expressed using the following equation: permeability index (%) = [(sample - negative control)/(positive control- negative control)] * 100.

### 3.5. Checkerboard Assay 

The combination effects between PYED-1 and gentamicin or amikacin against *S. maltophilia* K279a cells were assessed by a microbroth checkerboard assay [48]. Serial twofold dilutions were prepared in CA-MHB to ranch the final concentration of gentamicin or amikacin ranging from 0.25 to 128 μg/mL, and PYED-1 ranging from 4 to 1000 μg/mL. Fifty microliters of antibiotic (gentamicin or amikacin) was added to the rows of a 96-well microtiter plate in decreasing concentrations, and 50 μl of PYED-1 was added to the column in decreasing concentrations. Microplates were inoculated with 100 μl of *S. maltophilia* K279a suspension with a final concentration of 10^5^ × CFU/mL and incubated at 37 °C for 18 h. The optical density at 595 nm was measured by using a microplate reader (Bio-Rad Laboratories S.r.l.). The effect of the interactions of PYED-1 with each of the tested antibiotic was quantified by calculating the fractional inhibitory concentration (FIC) index as follows: FIC index = FIC of PYED-1 + FIC of antibiotic, where FIC of PYED-1 (or antibiotic) is the ratio of MIC of PYED-1 (or antibiotic) in combination and MIC of PYED-1 (or antibiotic) alone. The following intervals of FIC index were used to interpret the experimental outcome: ≤0.5, synergistic; >0.5 to ≤1.0, additive; >1.0 to ≤2.0, indifferent; and >2.0, antagonistic effects [48]. All experiments were repeated three times. 

### 3.6. Biofilm Assay

Biofilm quantification assays were performed in 96-well microtiter plates using a crystal violet (CV) method as previously described [46]. Briefly, overnight cultures of *S. maltophilia* K279a were diluted with fresh trypticase soy broth (TSB) with 0.5 % glucose to obtain a bacterial suspension of 5 × 10^6^ CFU/mL. One hundred of the bacterial suspension were added to 96-well sterile flat-bottom polystyrene plates in the presence of 100 μl of sub-MIC concentrations of PYED-1 ranging from 2 to 128 μg/mL. After 24 h incubation at 37 °C, the planktonic cells were gently aspirated, then the biofilms were washed twice times with sterile PBS (pH 7.2). Two hundred microliters of 0.1% crystal violet was added and incubated at room temperature for 15 minutes. Crystal violet was removed by pipetting, and wells were washed three times with 200 μl sterile PBS (pH 7.2). Plates were air-dried and 200 μL of ethanol was added. After 20 min, the biofilm biomass was quantified by measuring the optical density at 595 nm using a microplate reader (Bio-Rad Laboratories S.r.l.).

### 3.7. RNA

Total RNA was isolated from *S. maltophilia* K279a cells grown in in CA-MHB at 37 °C at 200 rpm to an OD_600_ of 0.4. Two mL of culture was subsequently treated with either PYED-1 at the concentration of 128 μg/mL or 0.016% DMSO and incubated at 37 °C at 200 rpm for 3 h. Two volumes (4 mL) of RNAprotect Bacteria Reagent was added to the cell suspensions and incubated for 5 min at room temperature. Next, the cell suspensions were centrifuged at 5000 × g for 10 min and the supernatant was decanted. RNA was purified according to the previously reported method [49]. RNA was quantified using a Nano-drop instrument (Thermo Fisher). 

### 3.8. RT-PCR

Total RNA was reverse-transcribed into cDNA using QuantiTect Reverse Transcription Kit (Qiagen), according to the manufacturer’s protocol. The RT-PCR was performed as previously described [50], using a SYBR Green master mix (Applied Biosystems). The oligonucleotides used in PCR experiments are reported in Table 5. 

The *rpo*B gene was used as the housekeeping control to normalize the expressions of genes of interest. RNA samples not treated with reverse transcriptase were routinely included as no template controls. Changes in transcript levels were determined using the 2^−ΔΔCT^ method [51]. RNA expression levels were determined by using three independent cultures, and all analyses were performed in triplicate.

### 3.9. Statistical Analysis

The calculation of arithmetic means and standard deviations was utilized to statistically analyze continuous variables. A t-test was used to determine statistical differences between treated and control groups for each dosage and each time point for the time–kill assay and for the biofilm assay. Statistical differences between treated and control samples expression values derived from RT-PCR were evaluated by using, for each gene, a Z-test on the null hypothesis that the average ΔΔCt values are equal to 1. All results were considered to be statistically significant at P < 0.05.

## 4. Conclusions

The results of the present study revealed that PYED-1 in combination with aminoglycosides, represent a significant tool to control *S. maltophilia* growth. Moreover, PYED-1 was identified as a promising agent for targeting biofilm and virulence of *S. maltophilia*. This might be a new strategy for the treatment of *S. maltophilia* biofilm-associated chronic infections.

## Figures and Tables

**Table 1:** MIC (μg/mL) and MBC (μg/mL) values of PD, DFZ, and DFZ precursors against *S. maltophilia* K279a.

Compounds	MIC	MBC
PYED-1	256	512
2 (DFZ)	>1000	>1000
3	750	>1000
4	>1000	>1000
5	512	>1000
6	>1000	>1000
7	1000	>1000
8 (PD)	>1000	>1000
STX	1	1

**Table 2:** MIC (μg/mL), MBC (μg/mL) values, and killing quotient (KQ) of PYED-1 against *S. maltophilia* K279a and *S. maltophilia* clinical isolates.

Strain	MIC	MBC	KQ	Strain	MIC	MBC	KQ
K279a	256	512	2	Sm0707	256	512	2
Sm0262	128	512	4	Sm0916	256	512	2
Sm0527	64	128	2	Sm1053	512	2000	4
Sm0528	128	256	2	OBGTC3	64	128	2
Sm0545	512	2000	4	OBGTC9	256	512	2
Sm0571	256	512	2	OBGTC20	128	512	2

**Table 3:** Synergistic effects of PYED-1 with antibiotics against *S. maltophilia* K279a.

Bacterial strain	Combination	MIC^a^ (μg/mL)	MIC^c^ (μg/mL)	FIC index
*S. maltophilia* K279a	PYED-1 /gentamicin	256/16	64/4	0.5
	PYED-1 /amikacin	256/16	64/2	0.375

^a^, MIC of one sample alone; ^c^, MIC of samples in combination; FIC index, fractional inhibitory concentration.

**Table 4:** RT-PCR analysis of biofilm and virulence factors gene expression in *S. maltophilia* K279a in the presence of PYED-1.

Gene			Fold Change ± SD	P Value
Smlt0648	*rmlA*	glucose-1-phosphate thymidylyl transferase	−1.10 ± 0.045 *	0.00002919
Smlt0686	*StmPr1*	extracellular protease	−2.21 ± 0.108 *	<0.0000001
Smlt0706	*smf-1*	fimbrial adhesin protein	−1.33± 0.144 *	0.00003563
Smlt1736	*hfq*	host factor-I protein	−1.20± 0.241	0.07368922
Smlt2120	*aph3’-IIc*	aminoglycoside 3’-phosphotransferase II	−2.93 ± 0.193 *	<0.0000001
Smlt2202	*smeZ*	multidrug efflux pump	−3.97 ± 1.381 *	0.00009759
Smlt2299	*fsnR*	response regulator protein	−2.03 ± 0.061 *	<0.0000001
Smlt3524	*sphB*	Serine-protease	−2.66 ± 0.067 *	<0.0000001
Smlt3615	*aac6′-Iz*	aminoglycoside 6’-N-acetyltransferase	−2.44± 0.486 *	0.00000015
Smlt3638		transmembrane hemolysin protein	−1.23 ± 0.083 *	0.00000143
Smlt4190	*sppA*	protease IV	−1.63 ± 0.088 *	<0.0000001
Smlt4209	*bfmA*	two component response regulator	−2.36 ± 0.174 *	<0.0000001
Smlt4395	*StmPr3*	Serine-protease	−2.90 ± 0.456 *	<0.0000001

* The asterisks indicate genes with a P value of <0.05.

**Table 5:** Oligonucleotide sequences used in this study.

Primer Name	Primer Sequence
aac6-Iz fw	TGTGGACTGATGCCGATG
aac6-Iz rv	GCACTTCAGCGAAACCAAC
aph3-IIc fw	CCGATCATGAAGACCTGGTG
aph3-IIc rv	GTCGATGAAACCGCTGAAAC
bfmA fw	AGTGAACTGCGCTTTTCTGG
bfmA rv	TGAATTCACCACGGCTGAG
fsnR fw	TCCTGATGGACCTGTCATTG
fsnR rv	TGCATGGTCATCATCACAAC
Hfq fw	TCTACAAGCACGCCATTTCC
Hfq rv	TACTCGTCTGCTTCATCACCTG
rmlA fw	TGCTGGGTGACAACATCTTC
rmlA rv	CCGGATCATTCACCCAATAG
rpoB fw	AGGAAATGCTGACGGTGAAG
rpoB rv	ACGAGCACGTTGAAGGATTC
smeZ fw	GCAGTGATGTACCTGTTTCTGC
smeZ rv	CAGCACATTGATCGAGAAGC
smf-1 fw	ACCGTGTCCAAGAACACTCTG
smf-1 rv	TGCACTTGGTCAGGTTGATG
Smlt3638 fw	GGTTGAAGGTATTCGACCACTG
Smlt3638 rv	ATCAGGGTGAACGGGGTATAG
sphB fw	CGCATCTTTCAGTCACCAAC
sphB rv	GTAATTGAAGTTGGCCAGCAC
sppA fw	AGTTTCTTCATCGGGCTGTG
sppA rv	ATGACGAACATCACCAGCAG
StmPr1 fw	GCCGAAGTCATCAACCTCTC
StmPr1 rv	ACACGTTGGTGTTGCTGTTG
StmPr3 fw	ATCGACAGCACCTGCAACTAC
StmPr3 rv	TTCACATCGCGATAGGACAG
